# Piezo1 Affects Temporomandibular Joint Osteoarthritis by Influencing pSmad3

**DOI:** 10.3389/fphys.2022.892089

**Published:** 2022-05-09

**Authors:** Chuan-Bin Wu, Tie Ma, Lin Ma, Qiang Wang, Qing Zhou

**Affiliations:** ^1^ Liaoning Provincial Key Laboratory of Oral Diseases, Department of Oral and Maxillofacial Surgery, School and Hospital of Stomatology, China Medical University, Shenyang, China; ^2^ Liaoning Provincial Key Laboratory of Oral Diseases, School and Hospital of Stomatology, China Medical University, Shenyang, China

**Keywords:** temporomandibular joint, osteoarthritis, interleukin-1 β, piezo1, pSmad3

## Abstract

**Objective:** The aim of this research was to study the expression of Piezo1 in a rat temporomandibular joint osteoarthritis animal model and to explore its mechanism for inducing inflammatory changes.

**Methods:** A total of 24male SD rats aged approximately 8 weeks were randomly divided into three groups: the blank control group, complete Freund’s adjuvant group (CFA), and CFA + inhibitor (GsMTx4) group. After 3 weeks, the condylar heads of the rats were evaluated by micro-CT, HE, immunohistochemistry, safranin O staining, and other experimental techniques. Protein was extracted from the subchondral bone, and the changes in Piezo1, Smad3, and pSmad3 levels in each group were detected by Western blotting. *p* < 0.05 was considered to indicate statistical significance.

**Results:** The degree of damage to the cartilage and subchondral bone in the Piezo1 inhibitor group was smaller than that in the CFA group. The expression level of Piezo1 in the CFA group was higher than that in the other groups, and the difference was statistically significant. The expression of pSmad3 in the CFA group was also higher than that in the other groups (*p* < 0.05).

**Conclusion:** Piezo1 is expressed in the condylar cartilage and subchondral bone of rats, and the degree of condylar destruction can be improved by influencing the pSmad3 expression.

## Introduction

Temporomandibular joint disorder (TMD) is becoming more common in clinical practice, and its incidence rate is as high as 28–88% (Jianlin et al., 2017; Shoukri et al., 2019). TMD refers to functional, structural, and organic changes of the temporomandibular joint and surrounding masticatory muscles caused by mental factors, psychosocial factors, abnormal occlusal function, trauma, minor trauma, immunity, sleep disorders, and systemic and local diseases (Fischer et al., 2020). According to the Wilkes–Bronstein staging system, temporomandibular disorders are divided into five stages (i.e., stages I-V) (Wilkes, 1989). Phases I and II involve reducible anterior displacement of the articular disc, and phase II has more pain symptoms than phase I. Phases III, IV, and V involve irreducible anterior displacement of the articular disc. Condylar bone destruction occurs in phase IV, and perforation of the articular disc (Acri et al., 2019) occurs in phase V.

Temporomandibular joint osteoarthritis (TMJ-OA) is a late manifestation of the temporomandibular joint disorder. Its symptoms include severe damage, resulting in symptoms such as difficulty chewing, limited mouth movement, and joint pain. TMJ-OA seriously affects the quality of life of patients (Ferendiuk et al., 2019; Salamon and Casselman, 2020; Ferneini, 2021; Andre et al., 2022).

Regarding medical history, most patients have poor oral behavior habits, such as lateral chewing, grinding teeth, and biting hard objects. Therefore, poor oral behavior is an important risk factor for TMJ-OA. Poor oral behavior habits create conditions under which the temporomandibular joint region suffers from unbalanced stress loading for a long time. Thus, in the temporomandibular joint, there should be some proteins that sense this stress. We proposed that stress-sensitive channel proteins have great significance for research on TMJ-OA. Long-term unbalanced stress loading activates stress-sensitive channel proteins, which in turn causes osteoarthritis.

As early as 2010, Coste et al. discovered the mechanically sensitive ion channel protein Piezo in mouse Neuro2A cell lines, and a series of *in vitro* experiments confirmed that Piezo1 was a mechanically sensitive ion channel that had never been reported. Since its discovery, Piezo1 has quickly attracted widespread attention from scholars in various fields (Fang et al., 2021; Jiang et al., 2021; Xu et al., 2021). The expression of Piezo1 as a stress-sensitive ion channel protein in TMJ-OA has rarely been reported.

In this article, we established an animal model of TMJ-OA in rats, established a control group and an inhibitor group to verify the expression of Piezo1 in TMJ-OA, and preliminarily explored its mechanism.

## Materials and Methods

### Experimental Animals and Treatment Groups

A total of 24 healthy Kunming male SD rats, approximately 8 weeks old and each weighing approximately 220 g, were randomly divided into three groups: the blank control group, the CFA injection group (25 µl CFA + 25 µl saline injected into each TMJ cavity), and CFA + GsMTx4 group (25 µl CFA +25 µl GsMTx4 injected into each joint cavity). Death occurred after 3 weeks.

### HE Staining

After obtaining the sample, it was cleaned with PBS and fixed with 4% paraformaldehyde. The tissue was rinsed with distilled water several times and soaked in 70% ethanol overnight and 80% ethanol, 90% ethanol, 95% ethanol, and 100% ethanol for 1.5 h each. The dehydrated tissue was soaked in xylene for 20 min. The cleared treated tissue was soaked in melted paraffin for 2 h. The tissue in the paraffin block was cut into 2.5-µm thick slices with a microtome and laid flat on an anti-devitrification slide. The slices were placed in a drying oven at 55°C so that the tissue slices were tightly attached to the anti-stripping slide. The paraffin sections were soaked in xylene I and xylene II for 10 min each. The dewaxed paraffin sections were soaked in 100% ethanol I, 100% ethanol II, 95% ethanol I, 95% ethanol II, 85% ethanol, and 75% ethanol for 3 min each. The sections were cleaned with double-distilled water three times for 2 min each time. The hematoxylin dye solution was added for 3 min. The excess staining solution was rinsed with tap water for approximately 3 min. Distilled water was used to wash the samples again. Decolorization with 1% hydrochloric acid alcohol was performed to remove excess hematoxylin staining solution from the cytoplasm. Eosin dye was added for 60 s. The excess dye solution was rinsed with tap water for approximately 3 min, and the samples were dehydrated with 75% ethanol for 10 s, 95% ethanol for 10 s, and absolute ethanol for 1 min. Xylene was used to clear the samples for 5 min. After the neutral resin was used to seal the samples, the nucleus was blue, and the cytoplasm was red or pink. Image acquisition was carried out.

### Safranin O Staining

Conventional dewaxing was performed. Freshly prepared Weigert dye solution was added for dyeing for 5 min. Acidic ethanol differentiation solution was added for 15 s. The samples were washed with distilled water for 1 min. The samples were then dipped in solid green dyeing solution for 5 min and washed with distilled water for 1 min. The sections were dipped and dyed with safranin O stain for 2 min and washed with distilled water for 1 min. The slices were washed with an acetic acid solution for 1 min to remove residual solid green and washed with distilled water for 1 min. The samples were dehydrated with 95% ethanol and absolute ethanol. Xylene was used for clearing, and the samples were sealed with optical resin.

### Immunohistochemical Staining

The samples were washed three times with PBS for 5 min each time; the samples were treated with 3% hydrogen peroxide at room temperature for 15 min. PBS was used for washing three times for 5 min each. Goat serum was added at room temperature for 60 min. The spin-dry sealing solution was removed, and the samples were incubated with the primary antibody at 4°C overnight. PBS was used to wash the specimens three times for 5 min each. The secondary antibodies were incubated with the samples in a working solution for 1.5 h at room temperature. PBS was used to wash the specimens three times for 5 min each. DAB was added, and the samples were protected from light for 10 min. The sections were washed twice with distilled water for 5 min. Hematoxylin was added for 5 min. Tap water was used to wash the samples until they were blue. One percent ethanol hydrochloride was used for differentiation for 5 s, followed by 95% ethanol dehydration for 2 min. The solution was changed to fresh 95% ethanol, and the samples were dehydrated for another 2 min. Xylene was used for clearing for 5 min. The solution was changed to fresh xylene, and the samples were cleared for 5 min. After the neutral resin was used to seal the samples, the nucleus was observed to be blue. Image acquisition of slices was performed.

### TRAP Staining

Paraffin sections (5 µm) were dewaxed and washed with water. Then, 1 ml of hexato-amine magenta was added to 18 ml of acetate solution, followed by 1 ml of purple phenol ASBI phosphate solution and 282 mg of potassium sodium tartrate. The pH value was adjusted to 5.0, and the solution was filtered for later use. Then, 1 ml of hexamine parabuchins was added to 18 ml of acetate for dissolution, and 282 mg of potassium sodium tartrate was added. The pH value was adjusted to 5.0, and the solution was filtered for later use. Slices were washed with distilled water at 37°C for 30 s. The solution was incubated with the samples at 37°C for 60 min. The samples were washed with deionized water for 3 min. Hematoxylin was added for 40 s. The samples were washed with tap water for 10 min and returned to blue. Glycerin gelatin was used to seal the samples.

### Western Blotting

The extracted protein was mixed with 5 × SDS loading buffer at a ratio of 4:1, and the protein was denatured by heating at 100°C for 6 min in a metal bath. Electrophoresis: a setting of 80 V was used to run the samples through the loading gel, and the voltage was changed to 120 V until bromophenol blue ran to the bottom of the gel without running off the gel. Membrane transfer: the clamp was opened to keep the black side level, and the sponge pad, filter paper, gel, PVDF membrane (activated by methanol), filter paper, and sponge pad were placed down in sequence. At the same time, the electrophoresis solution was replaced with a transfer solution, and the membrane transfer time was recorded. The membrane was removed, the front and back sides were marked, the membrane was washed in TBST for 1 min, and then, the membrane was closed at room temperature with 5% skim milk sealing solution for 1 h. After sealing, the membrane was washed with TBST three times for 5 min each time. The primary antibody was diluted 1:1000 with the primary antibody diluent and incubated overnight at 4°. The membranes were washed with TBST three times for 10 min each time, diluted with two antibodies, incubated at room temperature for 1.0 h, and washed with TBST three times for 10 min each time. The ECL exposure solution was evenly mixed according to liquid a: liquid B ratio of 1:1, evenly covered the whole film, and placed into an exposure instrument for exposure and detection for 1 min.

## Results

### Complete Freund’s Adjuvant Group + Inhibitor Reduces the Degree of Destruction of the Condylar Cartilage and Subchondral Bone

**FIGURE 1:**
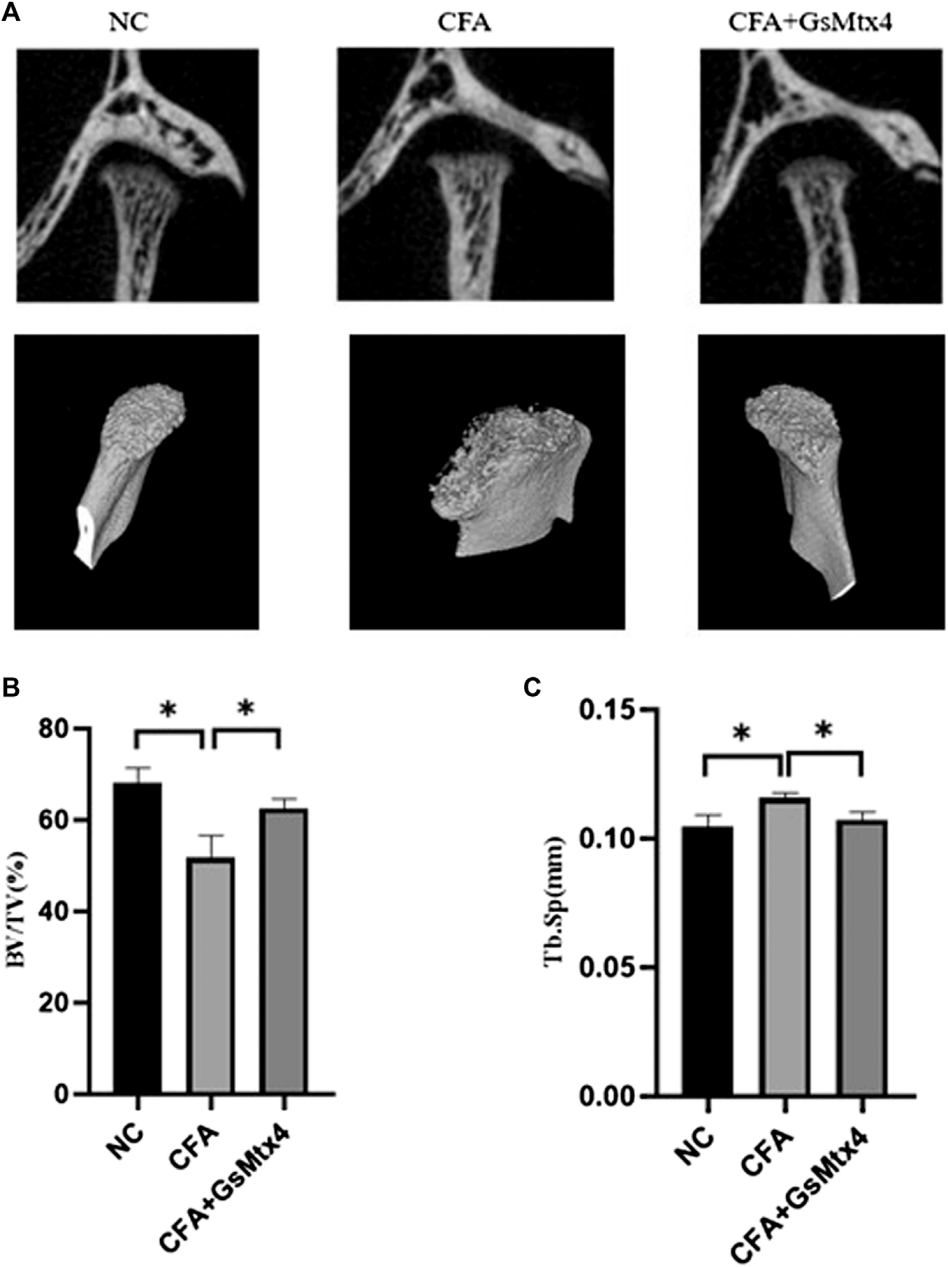
**(A)** Coronal position and 3D reconstruction image show that more condylar bone was absorbed in the CFA group than in the other two groups. **(B)** BV/TV: The bone volume fraction in the CFA group was lower than that in the other two groups, and the difference was statistically significant. **(C)** Tb.sp: The trabecular bone separation value in the CFA group was higher than that in the other two groups. *p < 0.05.

BV/TV decreased and Tb.sp increased in the CFA group, while BV/TV increased and Tb.sp decreased in the CFA + GsMTx4 group. The difference between the two groups was statistically significant (Figure 1).

**FIGURE 2:**
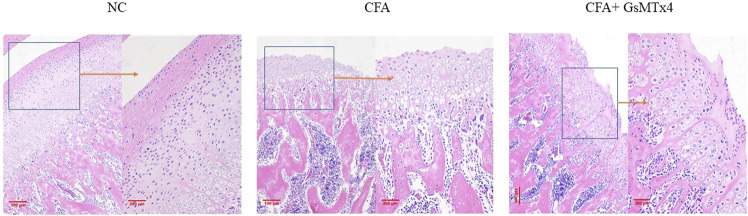
NC: clear cell level; CFA: damage to the surface zone and value-added zone of the condylar cartilage layer and damage to the exposed subchondral bone with a partial hypertrophy zone; CFA + GsMTx4: damage to the surface zone and value-added zone.

HE staining verified the micro-CT results. The HE section of the blank control group showed that the condylar chondrocytes of the rat were well-defined, without the phenomena of intercellular matrix degradation and cell-level disorder. In the CFA group, we found the destruction of the cartilage level, the degradation of the intercellular matrix, and cell-level disorder, with the disintegration of the cartilage layer and involvement of the subchondral bone. In the inhibitor group, the cartilage surface zone and the value-added zone were destroyed; however, the hypertrophy band was relatively complete (Figure 2).

**FIGURE 3:**
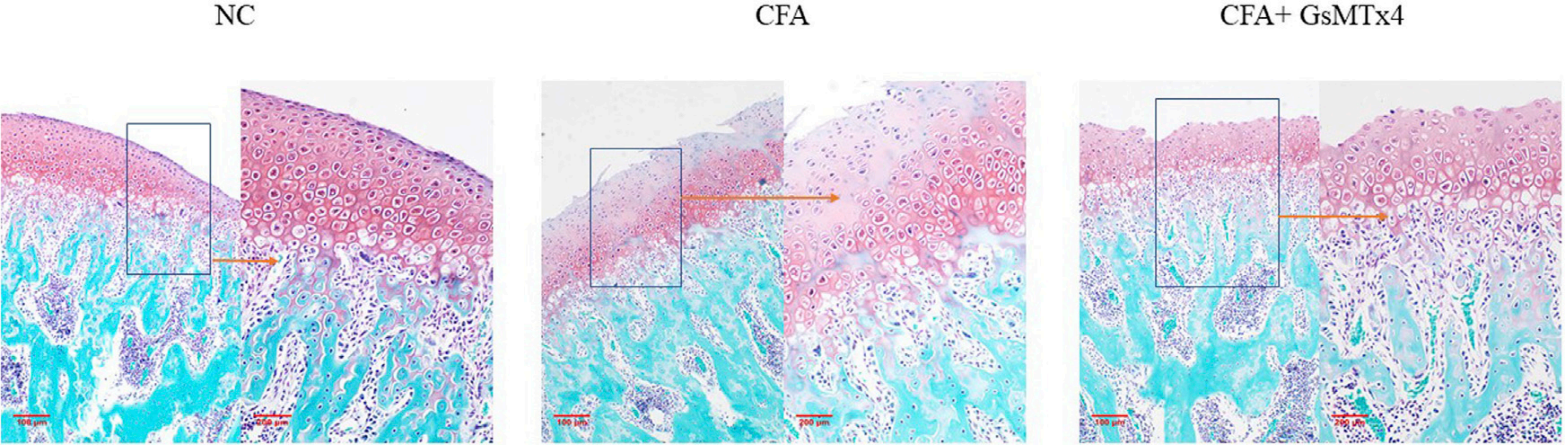
NC: clear cartilage level; CFA: damage to the cartilage surface zone and value-added zone, partial damage of the hypertrophy zone; CFA + GsMTx4: damage to the surface zone and value-added zone, with a relatively complete hypertrophy zone.

Safranin O staining showed changes in the cartilage after death at 3 weeks in the three groups of animals. The blank control group had a normal structure at the cartilage level. In the CFA group, cartilage-level destruction was observed, and the defect depression could be seen in some parts; the surface zone and value-added zone disappeared, and the hypertrophy zone was also involved. Although the surface zone was damaged in the inhibitor group, the hypertrophy zone was clear. It was further confirmed that GsMtx4 has an inhibitory effect on TMJ-OA (Figure 3).

**FIGURE 4:**
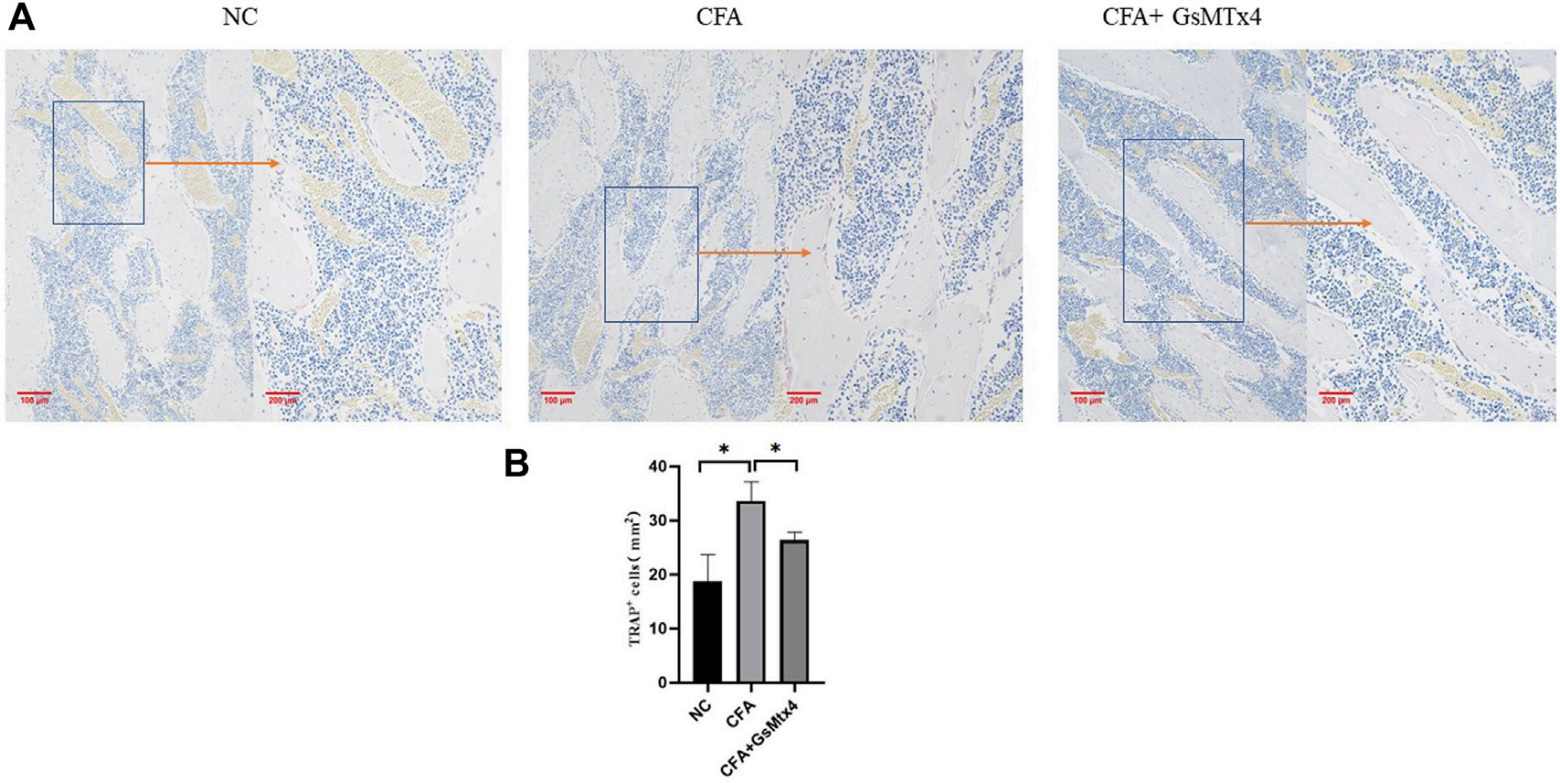
**(A)** Distribution of osteoclasts in each group; **(B)** Statistical analysis of the number of osteoclasts in each group. *p < 0.05.

The nucleus of osteoclasts was red-stained and distributed around the bone trabecula. TRAP staining results showed that the number of osteoclasts in the CFA group was greater than that in the other two groups, and the difference was statistically significant, *p* < 0.05. The number of osteoclasts in the inhibitor group was not significantly different from that in the normal control group (Figure 4).

### Piezo1 Affects Changes in the Condylar Bone and Subchondral Bone by Influencing pSmad3

**FIGURE 5:**
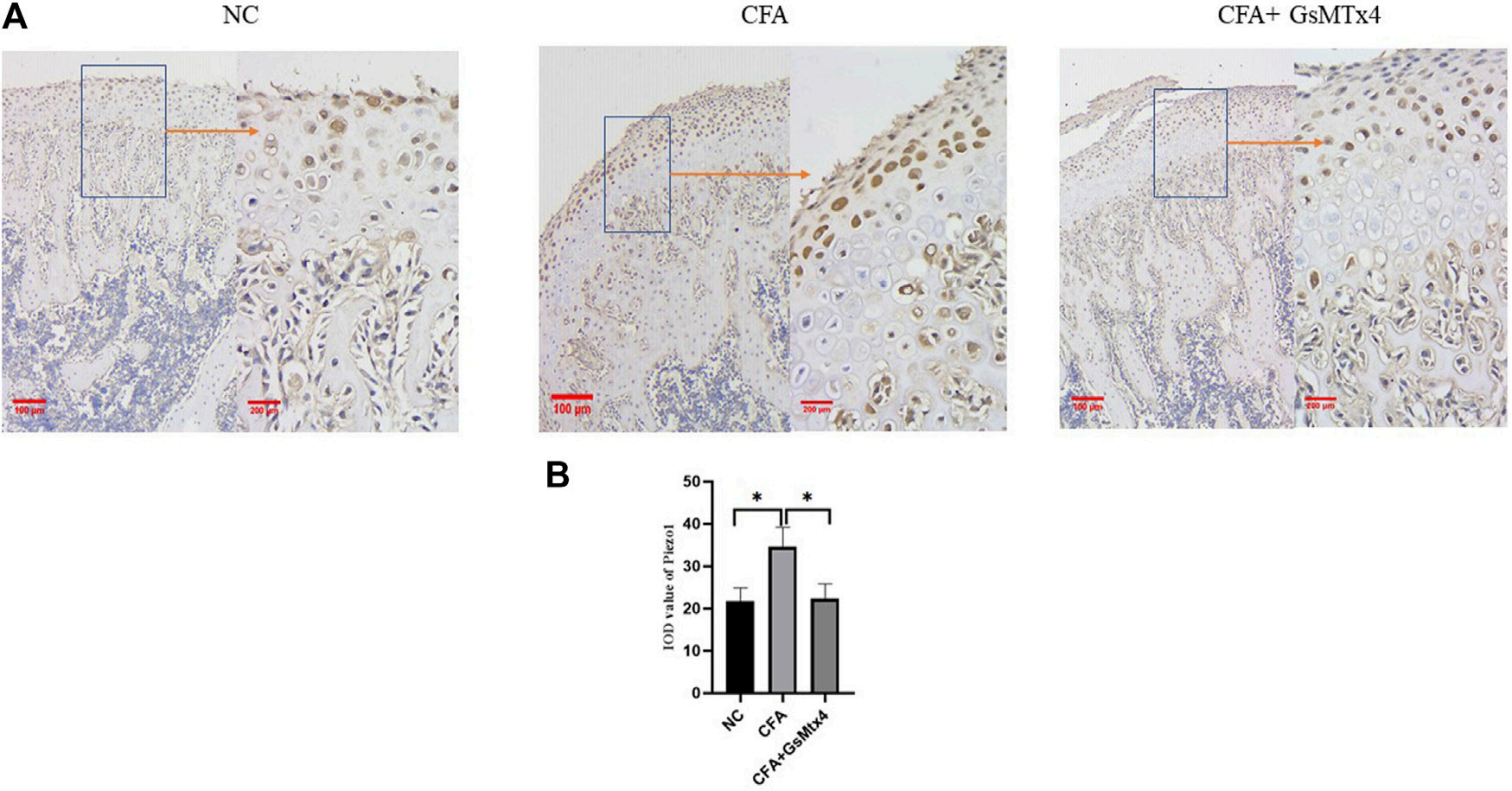
**(A)** Expression of Piezo1 in each group. **(B)** Statistical analysis of the Piezo1 expression in each group. *p < 0.05.

**FIGURE 6:**
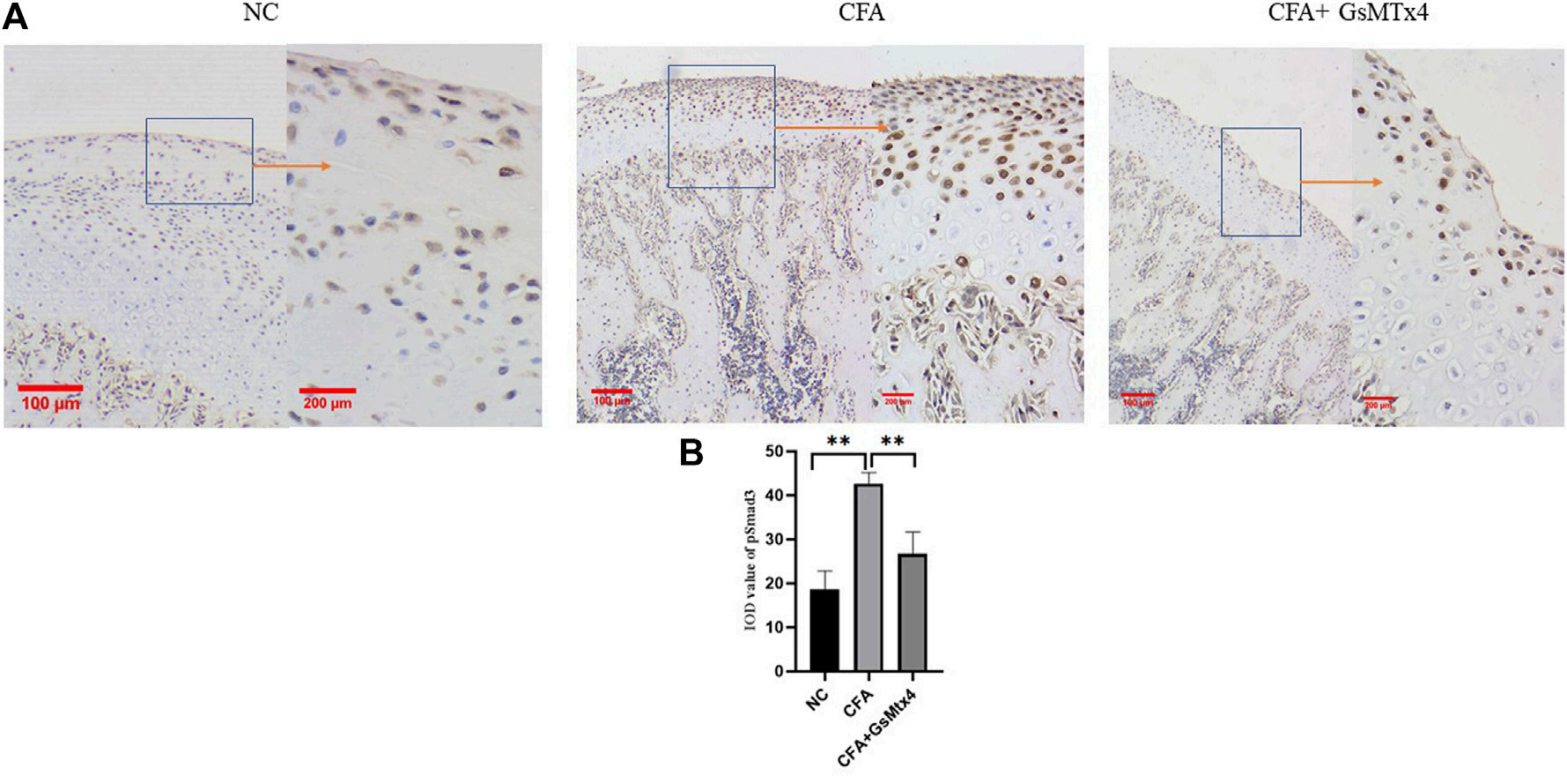
**(A)** pSmad3 expression in each group; **(B)** Statistical analysis of the pSmad3 expression in each group. **p < 0.01.

In animal models, we performed immunohistochemical staining for Piezo1 in each group. Piezo1 was expressed in the condylar cartilage and subchondral bone in the blank control group, and in the CFA model group, its expression was the highest. In the inhibitor group, the expression of Piezo1 decreased, and the difference was statistically significant (Figure 5). pSmad3 in the condyles of each group was immunohistochemically stained. It was found that pSmad3 was expressed in the condylar cartilage and subchondral bone. In the CFA group, its expression was higher than that of the other two groups, *p* < 0.05 (Figure 6).

**FIGURE 7:**
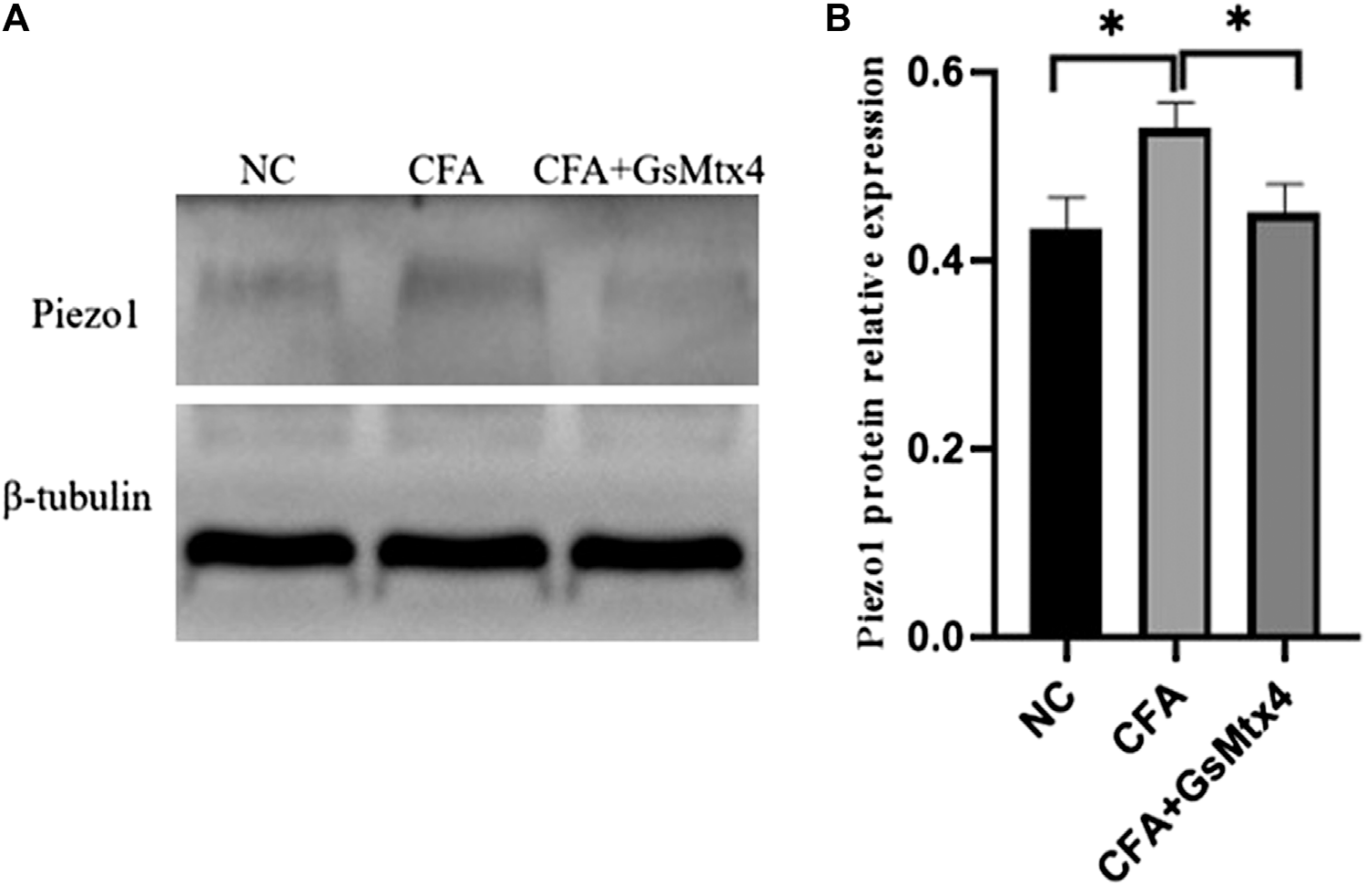
**(A)** Piezo1 expression in each group. **(B)** Statistical analysis of the Piezo1 expression in each group. *p < 0.05.

**FIGURE 8:**
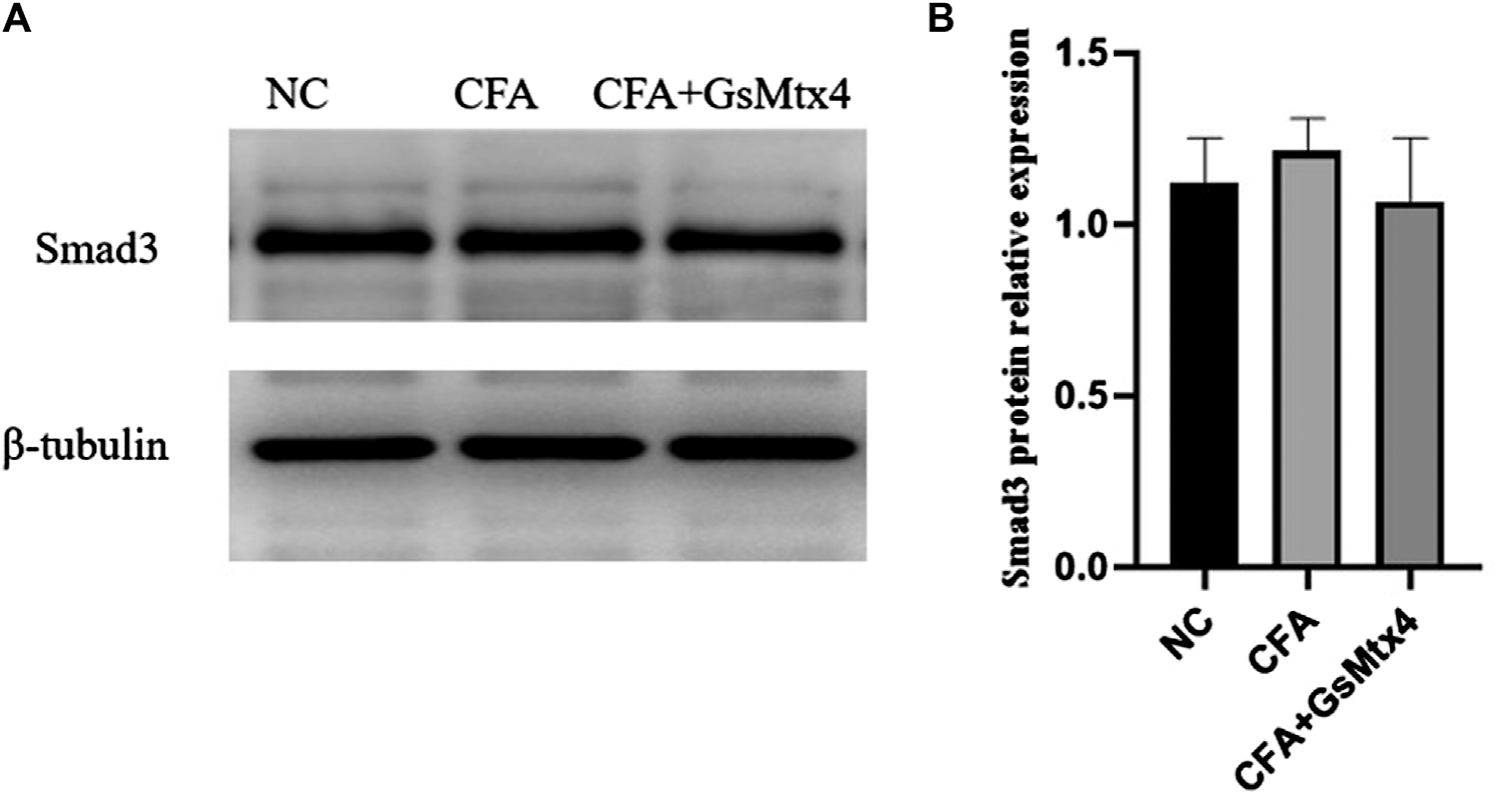
**(A)** Smad3 expression in each group; **(B)** Statistical analysis of the Smad3 expression in each group.

**FIGURE 9:**
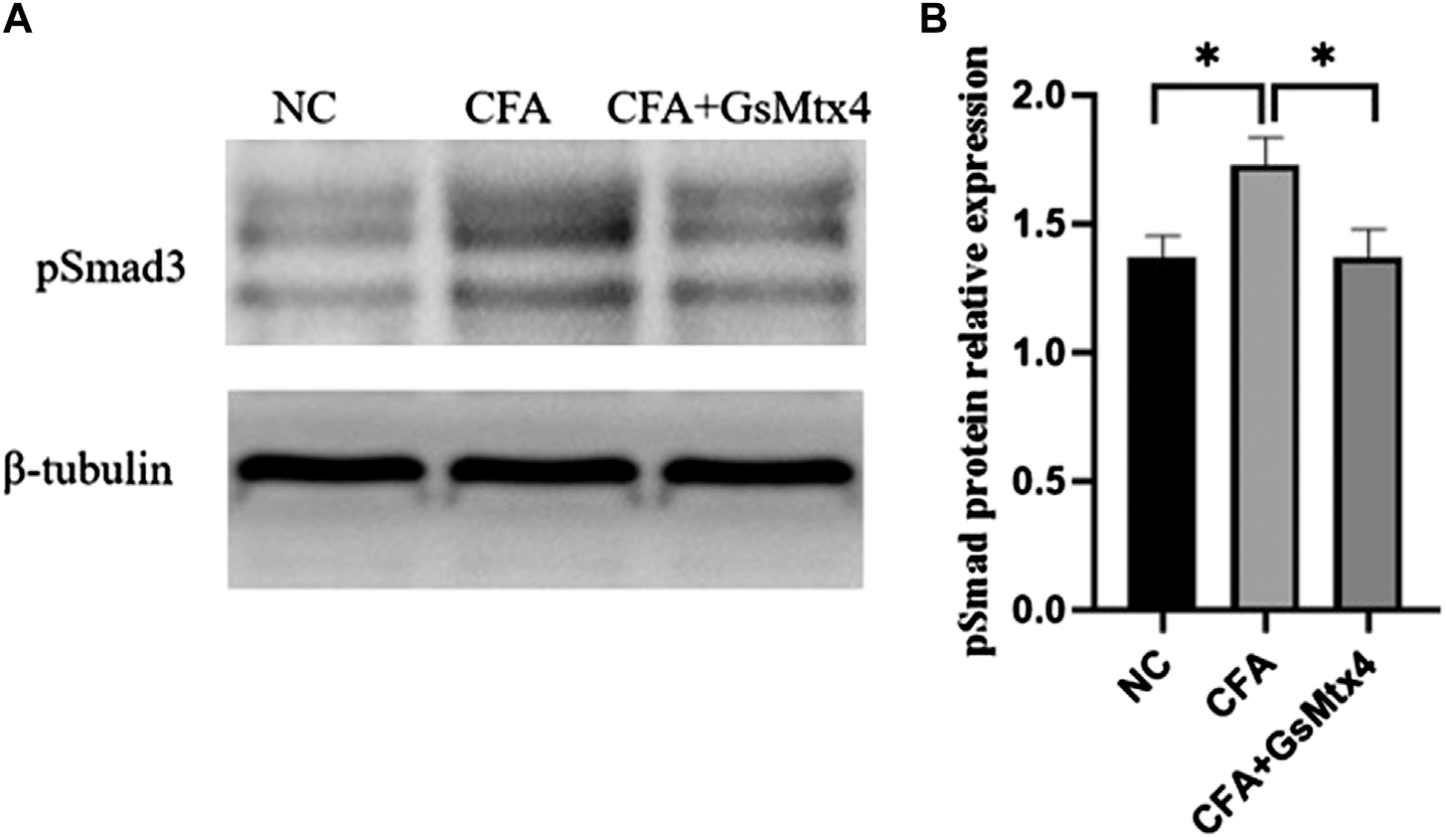
**(A)** pSmad3 expression in each group; **(B)** Statistical analysis of the pSmad3 expression in each group. *p < 0.05.

Next, we used WB to detect the protein expression of Piezo1, Smad3, and pSmad3 in the subchondral bone from the rats in each group. The expression of Piezo1 was the highest in the CFA group, and the difference was statistically significant compared with the other two groups (*p* < 0.05) (Figure 7). The difference in the expression of Smad3 in each group was not statistically significant, *p >* 0.05 (Figure 8). The expression of pSmad3 was the highest in the CFA group, and the difference was statistically significant compared with the other groups (*p* < 0.05) (Figure 9). From this, we concluded that in CFA-induced animal models of TMJ-OA in rats, the expressions of Piezo1 and pSmad3 were also increased. When the expression of Piezo1 was inhibited, the expression of pSmad3 also decreased.

## Discussion

The cause of temporomandibular joint osteoarthritis (TMJ-OA) is complex, and the structure of the temporomandibular joint is not simple. It is composed of a joint capsule, joint disc, synovium, cartilage, and subchondral bone (Arda et al., 2022). In TMJ-OA, any part can be affected, and the study of any component is also meaningful. At Oral Junior College Hospital, through X-ray or CBCT examination, we routinely focused on the changes in the condylar bone: is the front slope absorbed? Are bone spurs formed? Therefore, we selected the mandibular condyle as the research object.

There are usually animal models for disease research, and there have been many reports on the establishment of TMJ-OA models in the literature. Surgery destroys the physiological structure and removes the articular disc, all of which can lead to temporomandibular arthritis and subchondral bone remodeling. TMJ-OA can also be achieved by injection of chemical irritants, including sodium iodoacetate and complete Freund’s adjuvant (CFA). CFA is an antigen solution emulsified in mineral oil and consists of inactivated and dried mycobacteria. The CFA injection method is simple and can repeatedly induce temporomandibular arthritis (Emanuela et al., 2019; Morel et al., 2019).

We established a CFA inflammation model based on the following two points. First, Piezo1 is known to be activated by mechanical signals. However, it can be activated by inflammatory signals in some cases (Velasco-Estevez et al., 2020; Lee et al., 2021). Second, the injection volume of the upper cavity of the temporomandibular joint of unilateral rats is 50 μl, and these liquid volumes can also exert a certain pressure. This study showed that 3 weeks after CFA injection, the condylar subchondral bone of rats changed, mainly manifested as a decrease in bone volume fraction, an increase in trabecular bone separation (Tb.sp), and tissue structure destruction. These phenomena indicate that this study successfully constructed an animal model of TMJ-OA by CFA injection.

We chose 3 weeks as the only experimental time point based on the following two points. Temporomandibular joint osteoarthritis is a progressive disease, and any time point may become a time point for patients to come to the clinic, so research at any time point must be meaningful. Second, we only want to suggest an animal model of TMJ osteoarthritis. References have reported that there may be changes in the cartilage and subchondral bone in rats after 3 weeks of CFA injection (Liqin et al., 2016), and our experimental results are also consistent with the aforementioned changes.

Before selecting the method of inhibiting Piezo1, this study referred to a large number of research studies. The literature indicates that Piezo1 can be activated by Yoda1 and Jedi1/2 and can be inhibited by GxMTx4 and Dooku1 (Bailong, 2020; Valeria et al., 2021; Xue-Fei et al., 2022). GsMTx4 is a spider venom peptide that selectively inhibits mechanically sensitive Piezo ion channels (mechanosensitive ion channels, MSCs), especially cation channels. The discovery of the Piezo channel and the report of its sensitivity to the inhibitor GsMTx4 are important milestones in the study of nonselective cationic mechanically sensitive ion channels in normal physiology and pathogenesis. GsMTx4 has been used to study the functional role of cationic mechanosensitive channels, especially in muscle tissue. The sensitivity of the Piezo channel to double-layer stress and its mechanical sensitivity in heterologous systems are key aspects (Li et al., 2019; Ihara et al., 2021) to determining the mechanism of GsMTx4 action. Through a literature review, we found that GxMTx4 has a better inhibitory effect, so we chose to inject GxMTx4 to achieve the experimental goals.

Previous research by our research group showed that IL-1β can activate the TGF-β1/Smad3 signaling pathway in MC3T3-E1 cells and regulate IL-6 in the cell supernatant by changing the expression level of pSmad3. pSmad3 plays an important role in this signaling pathway (Xin et al., 2020). Can the change in the Piezo1 expression also regulate the whole inflammatory environment by changing pSmad3? In animal experiments, we used an injection of complete Freund’s adjuvant (CFA) to prepare animal disease models, injected GsMTx4 to inhibit Piezo1, and then used micro-CT, HE staining, safranin O staining, immunohistochemistry (Piezo1 and pSmad3), TRAP staining, and WB to detect the indicators.

In this study, in the animal disease model constructed by injection of CFA, after further injection of GsMTx4, it was found that GsMTx4 can alleviate the inflammatory destruction caused by CFA. After injection of GsMTx4, the bone volume fraction (BV/TV) value was significantly higher than that of the CFA group, while the trabecular bone separation (Tb.sp) value was decreased. The subchondral bone in the CFA injection group was largely destroyed, and the number of osteoclasts increased. The number of osteoclasts in the CFA + GsMTx4 group was smaller than that in the CFA injection group. Immunohistochemistry and Western blotting were used to detect the expressions of Piezo1, Smad3, and pSmad3. The results showed that the expressions of Piezo1 and pSmad3 increased after CFA treatment compared with those in the control group. However, after GsMTx4 treatment, the expressions of Piezo 1 and pSmad3 decreased compared with those in the CFA injection group. The WB experimental results also confirmed that Piezo1 can regulate the osteoarthritic environment by changing pSmad3 levels.

There are also some limitations to this study. The animals were euthanized 3 weeks after model establishment, and the regulatory effect of Piezo1 was not evaluated after a longer time point. However, this experiment was effective in pointing out the role of Piezo1 in TMJ-OA, which provides a new target for the conservative treatment of TMJ-OA.

## Data Availability

The raw data supporting the conclusions of this article will be made available by the authors, without undue reservation.
